# Targeting Mitochondria by Plant Secondary Metabolites: A Promising Strategy in Combating Parkinson’s Disease

**DOI:** 10.3390/ijms222212570

**Published:** 2021-11-22

**Authors:** Sajad Fakhri, Sadaf Abdian, Seyede Nazanin Zarneshan, Esra Küpeli Akkol, Mohammad Hosein Farzaei, Eduardo Sobarzo-Sánchez

**Affiliations:** 1 Pharmaceutical Sciences Research Center, Health Institute, Kermanshah University of Medical Sciences, Kermanshah 6734667149, Iran; sajad.fakhri@kums.ac.ir; 2 Student Research Committee, Kermanshah University of Medical Sciences, Kermanshah 6734667149, Iran; abdian.ph@gmail.com (S.A.); Nazanin.Zarneshan75@gmail.com (S.N.Z.); 3 Department of Pharmacognosy, Faculty of Pharmacy, Gazi University, 06330 Ankara, Turkey; esrak@gazi.edu.tr; 4 Department of Organic Chemistry, Faculty of Pharmacy, University of Santiago de Compostela, 15782 Santiago de Compostela, Spain; 5 Instituto de Investigación y Postgrado, Facultad de Ciencias de la Salud, Universidad Central de Chile, Santiago 8330507, Chile

**Keywords:** neurodegenerative disease, Parkinson’s disease, secondary metabolites, phytochemicals, mitochondria, signaling pathway

## Abstract

Parkinson’s disease (PD) is one of the most prevalent and debilitating neurodegenerative conditions, and is currently on the rise. Several dysregulated pathways are behind the pathogenesis of PD; however, the critical targets remain unclear. Accordingly, there is an urgent need to reveal the key dysregulated pathways in PD. Prevailing reports have highlighted the importance of mitochondrial and cross-talked mediators in neurological disorders, genetic changes, and related complications of PD. Multiple pathophysiological mechanisms of PD, as well as the low efficacy and side effects of conventional neuroprotective therapies, drive the need for finding novel alternative agents. Recently, much attention has been paid to using plant secondary metabolites (e.g., flavonoids/phenolic compounds, alkaloids, and terpenoids) in the modulation of PD-associated manifestations by targeting mitochondria. In this line, plant secondary metabolites have shown promising potential for the simultaneous modulation of mitochondrial apoptosis and reactive oxygen species. This review aimed to address mitochondria and multiple dysregulated pathways in PD by plant-derived secondary metabolites.

## 1. Introduction

Humans have always been affected by various neurological complications [1,2]. Neurodegenerative diseases greatly influence the central and peripheral nervous system and lead to the loss of both physical and mental functions [3,4]. Several mechanisms are behind neurodegeneration, including neuronal inflammation, oxidative stress, apoptosis, autophagy which cause cell death [1,3,5]. 

Considering the involvement of multiple dysregulated pathways in the pathogenesis of neurodegenerative diseases, revealing the critical signaling pathways in such disorders seems to pave the road for developing alternative therapies. Accordingly, mitochondria-associated disorders, as well as interconnected oxidative stress, and reactive oxygen species (ROS) seem to be common mediators involved in neurodegeneration [1,5]. In this line, targeting mitochondria-associated signaling pathways could open new roads in combating Parkinson’s disease (PD), ischemic brain injury (IBI), spinal cord injury (SCI), Alzheimer’s disease (AD), Huntington’s disease (HD), multiple sclerosis (MS) and amyotrophic lateral disease (ALS) [6,7,8]. 

Mitochondria are vital components of eukaryotic cells, and are responsible for producing cellular energy [7,8], especially for more active cells in the body [9]. Mitochondria supply this energy through the oxidative phosphorylation mechanisms by associated complexes [10]. In addition to the task of energy production, mitochondria are also involved in the procedures related to cell survival/death, as well as maintaining the balance of ions and cell metals [5]. These various functions in the mitochondria are performed by constituent elements in normal conditions of cellular equilibrium [8]. Under neuronal dysfunctionalities, the aforementioned cellular balance is lost, and mitochondrial function in neuronal cells is impaired [8,11]. In such a situation of neuronal damage, mitochondria encounter a dysregulation in ROS production and sensitivity to oxidative stress, leading to cell damage [12,13]. Functional and morphological changes in mitochondria and abnormalities in its enzymes lead to nerve damage and subsequent neurodegenerative diseases [8]. Accordingly, mitochondrial dysfunction is one of the causes of neurodegenerative diseases [5,8,12]. 

In neurodegenerative diseases, the mitochondria play critical roles in supplying energy and stability of nerve cells to prevent the destruction of dopaminergic neurons in PD. In addition, the role of mitochondrial toxins is also undeniable. For example, 1-methyl-4-phenyl-1,2,3,6-tetrahydropyridine (MPTP) blocked the activity of complex I in mitochondria, elevated ROS output, and increased the release of apoptogenic proteins, which ultimate causes cell death [14,15]. In addition, mitochondria have a close interconnection with several dysregulated pathways in PD. So, simultaneous targeting of mitochondria and close cross-talked mediators could pave the road for combating PD. 

There is no certain cure for PD and other NDDs, but various strategies have been employed to control associated symptoms by influencing dysregulated mechanisms [16]. Considering the higher level of side effects and lower efficacy of synthetic therapies in combating neuronal complications, natural products have drawn attention towards novel alternative therapies [17,18,19]. Consequently, plant-derived secondary metabolites are of great importance in modulating the mitochondrial disorders and interconnected inflammatory/oxidative pathways in neuronal disorders [8,19]. These secondary metabolites simultaneously attenuate mitochondrial function via several mechanisms, including the inhibition of mitochondrial complexes and ROS-related pathways, the suppression of apoptotic pathways, and preventing the production of α-synuclein misfolded aggregates [20,21].

A previous study reviewed the role of flavonoids in targeting mitochondria towards cytoprotection [22]. The involvement of phosphoinositide 3-kinases (PI3K)/protein kinase B (Akt) and nuclear factor erythroid-2-related factor 2 (Nrf2) is also highlighted in modulating mitochondrial activity during PD [23,24]. However, few potential alternative therapies have been introduced in the modulation of mitochondrial activities. This is the first review on the targeting of mitochondrial pathways by plant secondary metabolites and the modulation of pathological conditions in PD. 

## 2. Mitochondria in PD

PD is one of the most common neurological diseases with specific symptoms, including rest tremor, rigidity, and bradykinesia [8,25]. By increasing the average age of people, the number of affected patients is expected to increase [11,26]. The average age of patients developing PD is over 50 years, but considering the effect of various environmental and genetic factors, PD can occur at younger ages with different symptoms [26]. Symptoms include impaired bradykinesia, involuntary movement, stiffness and issues related to mental disorders [3,26]. Although numerous molecular mechanisms are engaged in the pathogenesis of neurodegeneration, synaptic destruction of dopamine and mitochondrial dysfunction are major players [8]. The fundamental neuropathology of PD is characterized by the gradual depletion of dopaminergic neurons in the substantia nigra pars compacta (SNc), as well as the existence of Lewy bodies (LBs) and Lewy neurites (LNs) [26]. These proteins originated from α-synuclein, a presynaptic protein in neuronal cells, and the destruction of dopaminergic neurons [25], involved in familial and intermittent PD. To perform their function, neurons need to supply energy provided by mitochondria [8,27]. In addition, mitochondria also play a vital role in maintaining the stability of nerve cells. So, by disrupting the mitochondrial-associated mechanisms, nerve cells encounter damage, followed by the occurrence of neurodegenerative diseases, especially PD [8,12]. 

In detail, during PD, dysregulation of the mitochondrial electron transport chain (ETC) and complexes I, II, III, IV, V leads to changes in cell energy, increases oxidative stress, causes mutations in mitochondrial genome, causes DNA deformities and alters the genes involved in PD [28]. Regarding the role of mitochondrial complexes, five enzymatic subsets are involved in the energy production of the oxidative phosphorylation system in mitochondria through electron migration. The complexes are named complex I (ubiquinone oxido reductase or NADH dehydrogenase), complex II (succinate dehydrogenase), complex III (cytochrome c-oxido-reductase or cytochrome bc1), complex IV (cytochrome c oxidase), and complex V (ATP synthase) [25]. Mitochondria have three enzyme complexes that produce different amounts of ROS [12]. Complex I, or NADH CoQ reductase, is one of the most important primary producers of ROS in the mitochondria. Changes in the structure of this complex and oxidative mediators are involved in developing PD [29]. Blockers of complex I mitochondrial ETC cause PD in people and animals, and knockouts of parkin or DJ-1, which are linked to hereditary PD, demonstrate impaired mitochondrial activity [30]. Inhibitors that interfere with the function of complex I, such as rotenone and MPTP, lead to PD. MPTP markedly lowered dopamine and its metabolites, which include 3,4-dihydroxyphenylacetic acid (DOPAC) and homovanillic acid (HVA) in the striatum [15]. MPTP creation in PD is caused by mitochondrial abnormalities. It involves a sequence of incidents such as inner mitochondrial membrane (IMM) depolarization, damaged oxidative phosphorylation, elevated ROS output, mitochondria matrix swelling, IMM cristae unfolding, loss of Ca^2+^ homeostasis, and the release of apoptogenic proteins via the outer mitochondrial membrane (OMM), which finally results in cell death [14]. Complex III, similarly to complex I, is the source of ROS in the mitochondria [12,13]. In this line, the PD-associated toxins MPTP, rotenone, pyridaben, trichloroethylene, and fenpyroximate interfere with the normal functioning of mitochondria enzymes/complexes to cause damage to neuronal cells [25].

During the procedure of mitochondrial energy production, ROS are also produced, which affects the normal function of neuronal cells [25]. Additionally, the mutation in mitochondrial and nerve cell genomes is another associated disruption mechanism. Accordingly, mutations of 12SrRNA and G11778A in mitochondrial DNA followed by changes in genes involved in PD in neuronal cells such as PINK1 (PTEN-induced kinase 1), PARK2 (parkin), DJ-1, and LRRKS (leucine-rich repeat kinase) lead to ROS production. This process is due to the imbalance between mitochondria and ROS, as well as the loss of cell protection against oxidative stress, autophagy of mitochondria and increase in the rate of α-synuclein [8,25]. 

Among the other mechanisms, abnormalities in mitochondrial fission and fusion play significant roles in PD. While mitochondrial dysfunction is well-known in PD, the roles of mitochondrial fission, Drp1, and fusion imbalance remain unknown. Irregular mitochondrial interactions with a Drp1 protein (a fission protein involved in mitochondrial fragmentation) are responsible for mitochondrial fission, and associated dysregulated proteins engaged in PD [8]. Enhanced production of Drp1, on the other hand, prevents muscle dysfunction. In addition, PINK1 mutations have been linked to inherited PD. Mitochondrial fission was stimulated by the PINK1/parkin pathway, and associated mutants dysregulate mitochondrial and tissue stability through decreasing mitochondrial fission. Mutations in the proteins α-synuclein, parkin, PINK1, and DJ1 correlate with neuronal mitochondrial malfunction in PD [8]. In SH-SY5Y cells, knockdown of PINK1 balance caused disruptions in mitochondrial activity and autophagy, which were all overturned when an RNA interference (RNAi)-resistant plasmid for PINK1 was reintroduced. In addition, mitochondrial morphological changes are caused by PINK1 mutations and mitochondrial fusion promoters, mitofusin 2 (Opa1) and fusin 1 (Mfn2) [8]. 

LRRK2, EIF4G1, VPS35, and PARK7 are other genetic-related factors playing critical roles in PD. A30P, E46K, and A53T missense mutations in α--synuclein enhance the proclivity of α-synuclein protein to construct β-sheets, which combine and form LBs. Several instances of this mutant gene have been found in various inherited cases of PD [26]. In addition, other factors predisposing a person to PD include toxins and iron deficiency in meals [26].

Nrf2 is a crucial modulator of detoxifying genes that help the body to withstand oxidative stress [15]. Surprisingly, new research suggests that the production of polo-like kinase 2 (PLK2) promotes antioxidant signaling by phosphorylating glycogen synthase kinase 3 (GSK-3β), boosting the nuclear transfer of Nrf2, thereby activating antioxidant response elements (ARE). This PLK2 might play an essential role in modulating mitochondrial function [15]. 

Levodopa is one of the primary drugs used in PD, which works to supply dopamine as its metabolic precursor [31]. In addition to levodopa and other conventional therapies, the focus on natural products and secondary metabolites has also increased [19,25,31]. Conventional treatments in PD are based on the mechanism of disruption, supply, and storage of dopamine in neurons [31]. In recent years, plant secondary metabolites are critical alternative therapies in combating neurodegenerative diseases, focusing on PD. 

## 3. Plant Secondary Metabolites and Mitochondria

As previously mentioned, plant secondary metabolites have different properties regarding controlling and treating various neurological diseases [19]. The use of secondary metabolites could interfere with the mechanisms modulating nerve cells dysfunctions [13]. From the mechanistic point of view, these metabolites have shown various antioxidant, anti-inflammatory, and anti-apoptotic effects interconnected to mitochondrial events in controlling and preventing neurodegenerative diseases [26]. There is also a significant relationship between the consumption of these metabolites and improving neurodegenerative diseases by targeting mitochondria [21]. 

**Figure 1:**
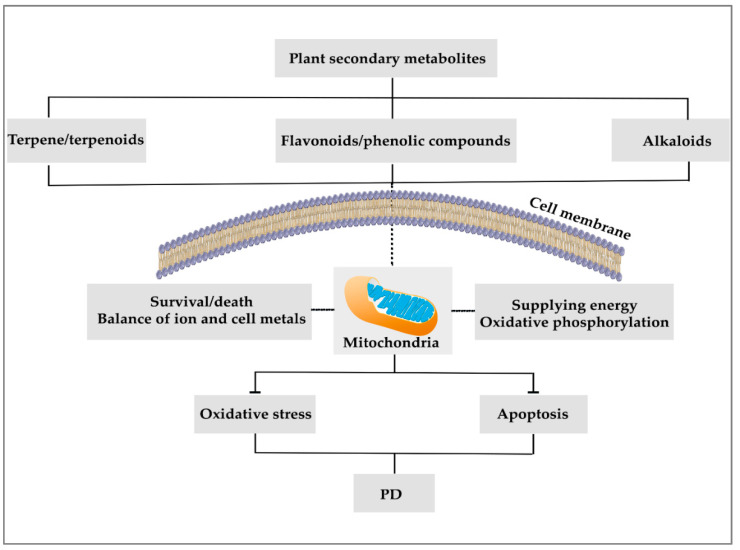
The potential of phytochemicals in the attenuation of mitochondria-related functions.

The efforts to protect the mitochondria and prevent the risk of apoptosis and oxidative stress are affected by the ROS-related pathways of phytochemicals [18,32] (Figure 1). Plant secondary metabolites also have antioxidant and modulatory effects on the mitochondria complex/enzymes of nerve cells by stimulating mitochondrial biosynthesis via the sirtuin1 (SIRT1), peroxisome proliferator-activated receptor gamma coactivator 1-alpha (PGC-1α), transcription factor A, mitochondrial (TFAM), and Nrf1 pathways, blocking mitochondrial fission [33], and maintaining its membrane stability [32]. 

## 4. Modulation of PD by Phytochemicals through Targeting Mitochondria

Secondary metabolites have shown promising effects on neurodegenerative diseases, specifically on PD, by targeting the mitochondria [19,25,34]. PD is a complex neurodegenerative disease characterized by mitochondrial dysfunction, oxidative stress, and neuroinflammation. In this line, the effects of polyphenols, terpene/terpenoids, and alkaloids are highlighted on the mitochondria in PD through various mechanisms.

### 4.1. Polyphenols Effects on Mitochondria in PD

Polyphenols are key secondary metabolites in modulating the mitochondria during PD. These secondary metabolites have a structure based on phenyl rings, including four main categories of flavonoids, lignans, stilbenes, and phenolic acids [6,13,25]. Flavonoids, in turn, have seven subunits that include flavonol, flavone, flavanone, flavanonol, flavanol, anthocyanin, and isoflavone [13,35]. Polyphenols prevent the production of α-synuclein misfolded aggregation, as well as mitigating oxidative stress, apoptotic and inflammatory processes caused by mitochondrial malfunction [21]. 

Baicalein is a leading active flavone element of the root of *Scutellaria baicalensis* and decreases the mitochondrial malfunction in an in vivo and in vitro experiment induced by 6-hydroxydopamine (6-OHDA) [36,37]. In an isolated brain mitochondrion, baicalein treatment reduced rotenone-induced ROS generation, ATP shortage, and mitochondrial swelling. Remarkably, baicalein increased mitochondrial respiratory action in isolated mitochondria. These findings imply that baicalein is a proper antioxidant with mitochondrial targeting with preventative properties against rotenone-induced neurotoxicity [38]. 

Puerarin, an isoflavone gained from *Pueraria thomsonii*, showed therapeutic effects on mitochondria through reducing the toxicity of 1-methyl-4-phenylpyridinium (MPP^+^)-induced in vitro model of PD [36]. In PC12 cells and primary rat midbrain neurons, puerarin reduced nitric oxide (NO)-mediated neurotoxicity in PD via upregulating mitochondrial enzyme arginase-2 for the selective regulation of mitochondrial dysfunction [39]. Additionally, the malfunction of the ubiquitin–proteasome system in the neuron has been extensively established as exacerbating PD. Puerarin also prevented apoptosis in MPP^+^-induced SH-SY5Y cells by increasing cell survival, enhancing morphological alterations, and decreasing apoptotic rate by modulating the ubiquitin–proteasome system [40].

In another study, flavonoids indicated therapeutic potentials on mitochondria enzyme complex I (e.g., luteolin, fisetin, robinetin, myricetin, rhamnetin and baicalein) and III (e.g., hispidulin and eupafolin) [13]. In addition, flavonoids defended against dopamine depletion and ROS initiation in PD [25]. 

Polyphenols substances such as quercetin and resveratrol in red wine also showed therapeutic potential on mitochondria by inhibiting apoptosis [13,20]. Resveratrol treatment also restored rotenone-induced mitochondrial membrane potential, altered mitochondrial dynamics, and elongated fragmented mitochondria in SH-SY5Y cells [41]. Quercetin also played essential roles in promoting mitochondrial biogenesis [42]. In SH-SY5Y cells exposed to MPP^+^ or lipopolysaccharides (LPS), pretreatment with quercetin substantially reduced mitochondrial damages. Tyrosine hydroxylase and mitochondrial controlling proteins were both upregulated by quercetin [43]. As previously described, PD is caused by mitochondrial malfunction and poor mitophagy. In a 6-OHDA-treated PC12 cells, quercetin administration enhanced mitochondrial quality control, decreased oxidative stress, and raised the levels of mitophagy markers. Furthermore, in rats with PD, quercetin alleviated the 6-OHDA-induced progression of PD-like motor abnormalities, decreased neuronal death, and reduced mitochondrial dysfunction [44]. Further research suggested that quercetin’s analysis was assumed to repair mitochondrial ETC abnormalities and upregulated. This activity is the foundation of neuroprotection shown in the parkinsonism caused by mitochondrial neurotoxin [45]. Hyperoside, a quercetin 3-o-galactoside, is a flavonolglycoside which reduced mitochondrial apoptotic signaling in PC12 cell in an in vitro experiment [46]. 

Silibilin, a flavonolignane extracted from *Silybum marianum*, has shown modulatory roles on mitochondrial membrane potentials (MMPs) in an in vivo experiment on a MPTP-induced PD model in mice [47]. Silibinin’s neuroprotective mechanism includes a decrease in mitochondrial damage and strengthening of the oxidative defense system. Dopaminergic nerve protection is achieved by stimulating mitophagy, which removes the detrimental consequences of damaged mitochondria. These data implied that silibinin has the potential to be further explored as a treatment option for PD [48]. In another study, silibinin significantly reduced the motor impairment and dopaminergic neuronal degeneration induced by MPTP. The findings suggest that silibinin has such benefits in MPTP-induced models of PD achieved through increasing the stability of mitochondrial membrane potential [49]. Another member of the lignan family, schisandrin, lessened ROS levels, decreased Ca^2+^’s impact and recovered mitochondrial membrane permeability capacities [25]. 

Naringenin, a flavanone, showed a regulatory effect in neurons and alleviated the function of mitochondria. This effect was exerted through increasing MMP and reducing ROS via affecting the Nrf2/ARE pathway in an in vitro experiment on the brains’ neurons of Sprague Dawley rats [50]. In addition, naringenin inhibited the mitochondria-related bioenergetics and redox dysfunctions caused by methylglyoxal in human neuroblastoma SH-SY5Y cells via the Nrf2/GSH pathways [51]. Naringin, a glycosylated naringenin, has been shown to defend against PD in animal models. In the striatum and substantia nigra pars compacta (SNpc) of rats, naringin decreased rotenone-induced dopaminergic toxicity. Subcellularly, naringin reduced the rotenone-induced reduction in mitochondrial function, stability, and bioenergetics in the animals’ SNpc, via a Nrf2-mediated path [52]. 

Isoliquiritigenin is a chalcone flavonoid gained from *Glycyrrhizae uralensis*. Isoliquiritigenin pretreatment fully inhibited the production of ROS as well as the dissipation of MMP and the presence of cytochrome c in the cytoplasm [53]. Inducers of the Nrf2/ARE pathway, such as isoliquiritigenin from licorice, showed the potential preservation of mitochondrial function in oxidative stress and neurodegenerative disease models, and also provided a unique strategy to prevent and treat aging-related neurodegenerative disorders, especially PD [23]. 

Phenolic acids, a subset of phenols, including ellagic acid and ferulic acid, affected mitochondria and thereby protected them from ROS-related pathways [32]. Protocatechuic aldehyde is another phenolic acid gained from the root of *Salvia miltiorrhiza*, which has a protective effect on mitochondria by blocking ROS production and keeping the activity of complex I in MPP^+^-incubated SH-SY5Y cells evaluated in an in vitro experiment [15]. In this line, another phenolic acid, caffeic acid, obtained from tea, wine, coffee, etc., blocked the production of ROS and normalized the activity of mitochondria in the 6-OHDA-induced SH-SY5Y cellular model of PD [54]. In the MPTP animal model of PD, caffeic acid’s phenethyl ester reduced dopaminergic neurodegeneration and dopamine loss. Furthermore, it reduced MPP^+^-induced neurotoxicity in vitro and effectively prevented MPP^+^-induced mitochondrial cytochrome c and apoptosis. As a result, caffeic acid could be helpful in delaying or stopping the progression of PD [55]. 

The major antioxidants contained in virgin olive oil are phenolic products, and in terms of quantity, the class of secoiridoids is the most abundant in olive oil [6]. Oleuropein and ligstroside are two major secoiridoids found in olive oil and can product against hydroxytyrosol and tyrosol. Oleuropein decreased the rate of superoxide anion in mitochondria [56]. In dissolved cells of the brain from mice, hydroxytyrosol prevented mitochondrial membrane potential depolarization and reduced the functions of mitochondrial complexes I, II, and IV. Tyrosol showed a defensive function against MPP^+^ in dopaminergic neurons [6]. In line with that, green and black tea polyphenols have incredibly strong antioxidant-radical scavenging effects on membrane fragments of mitochondria in the brain [18]. Tea polyphenols could preserve dopamine neurons by dramatically suppressing DA-related damage, inhibiting DA oxidation, conjugating with DA quinones, scavenging ROS, and modulating the anti-oxidative signaling pathways Nrf2-Keap1 and PGC-1α [57]. In line with the modulation of Nrf2, pinocembrin, a flavonoid in propolis, provided mitochondrial and cellular safety by suppressing ERK1/2 and attenuation of Nrf2 [58].

Curcumin, a polyphenol gained from *Curcuma longa*, protected mitochondrial function in PD by blocking the activity of MPP^+^ and ROS production in an in vivo and in vitro experiment [36,42,59] and preventing mitochondrial membrane damage in PD [18,19,34]. Targeting the exact mechanism, vanillin, a phenolic aldehyde, reduced rotenone-induced ROS production and mitochondrial malfunction in SH-SY5Y cells [54]. As another phenolic compound, luteolin, obtained from the ripe seed of *Perilla frutescens*, also played a preventive role in ROS production and maintained mitochondria’s activity at a normal level [46,60]. Another phenolic compound, epigallocatechin gallate (EGCG), a type of catechin obtained from green tea, inhibited the mitochondrial malfunction via the stimulation of the AMP-activated protein kinase (AMPK) pathway [61]. 

**Figure 2:**
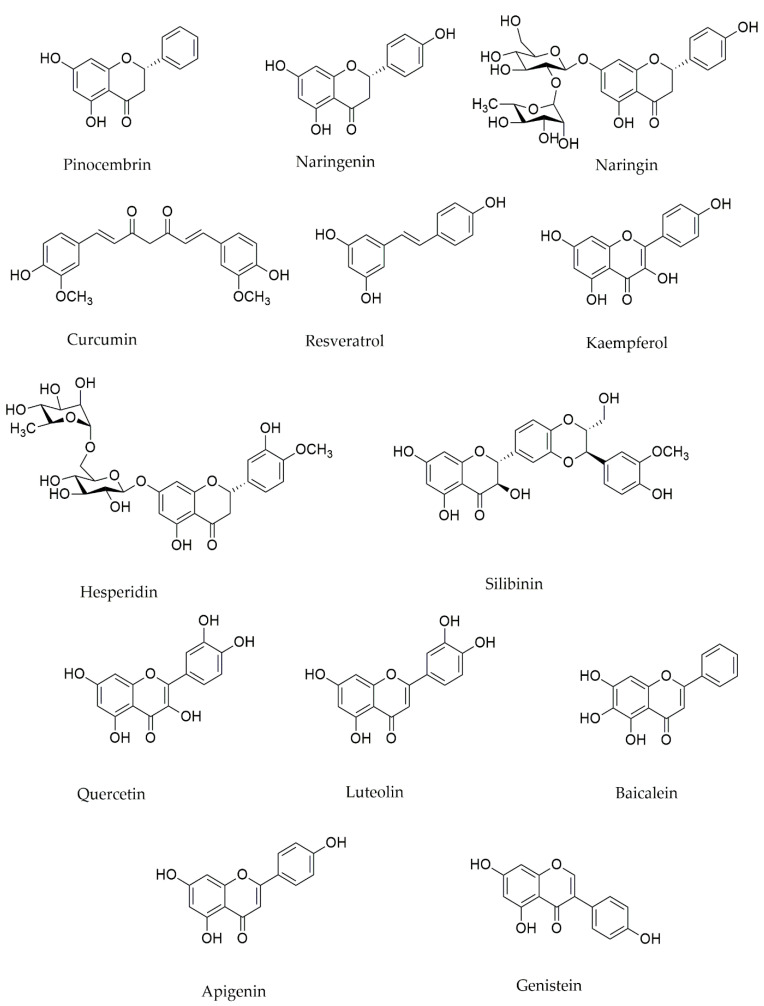
The chemical structure of flavonoids/phenolic compounds.

Hesperidin, a flavonoid glycoside often observed in citrus fruits, showed a protective effect on mitochondria by modulating mitochondrial complex I, IV, and V in an in vivo experiment on mice [62]. The chemical structures of flavonoids/phenolic compounds are provided in Figure 2. 

Therefore, several studies asserted that flavonoids and phenolic compounds might be useful in the prevention/treatment of PD by targeting mitochondria and related apoptotic/oxidative mediators, including MMP, ROS, Nrf2/ARE, NO, JNK, p38MAPK, Bcl-2/Bax ratio, PI3K/Akt, nuclear factor Kappa-B (NF-κB), PGC-1α and the activity of complexes I, II, and IV.

### 4.2. Alkaloids and Mitochondria in PD

Alkaloids are primary phytochemicals with nitrogen atoms, usually as part of a cyclic system or in combination. This class of phytochemicals affect social life, economic status, and health aspects of human life in various diseases, especially PD [63,64]. Nicotine is a naturally occurring alkaloid found in *Nicotiana tabacum* roots and leaves. It is also predominantly present in smaller quantities of various kinds of the Solanaceae family, such as potatoes, tomatoes, eggplants, and peppers chiles. Nicotine inhibits ETC from NADH to complex I, blocks the action of the NADH-ubiquinone reductase, resulting in a measurable reduction in oxygen intake by mitochondria. By suppressing complex I, nicotine resulted in less electron depletion and, as a result, less ROS generation [65]. In another study, nicotine interfered with the mitochondrial apoptotic pathway and shielded neurons against apoptosis caused by oxidative stress [32]. As provided by Xie et al., nicotine has been linked to a lower chance of acquiring PD. Nicotine suppressed MPP^+^ and calcium-induced mitochondrial high-amplitude swelling, and also cytochrome c release from intact mitochondria in SH-SY5Y cells. Their results indicate that nicotine showed a receptor-independent neuroprotective impact during PD by attenuating MPP^+^, calcium-induced mitochondrial, and cytochrome c [66]. Another report demonstrated that nicotine mediated mitochondrial dynamics and impacted mitochondrial affiliation from microtubules, greatly increased IP3 receptor clustering and modulated mitochondria-endoplasmic reticulum communication, as well as increasing mitochondrial biogenesis in cultured hippocampal neurons [67]. Nicotine has also shown mitochondrial impacts at small doses in vivo and in vitro, maintaining mitochondrial function in the rat CNS [68]. According to these findings, nicotine inhibited H2O2-induced astrocyte apoptosis, through the mitochondrial route by stimulating α7 nicotinic acetylcholine receptors (α7-nAChRs), and astrocytes which have been linked to immunological responses are associated with PD. In addition, considering the ability of α7-nAChRs expressed in astrocytes, it might be a potential therapeutic approach for neurodegenerative diseases [69].

Caffeine (1,3,7-trimethylxanthin) is a naturally occurring alkaloid found in coffee, tea, and cocoa plants [70]. Caffeine can block ROS production and enhances the activity of mitochondria. It also increases mitochondrial formation by stimulating Nrf2-keap1 and PGC-1α implicated in mitochondrial biogenesis, as well as having interconnected antioxidant and anti-inflammatory mechanisms. Additionally, caffeine reinstates transcription genes engaged in various activities such as cell death, cell cycle control, oxidative stress, and the expression of mitochondrial function-related genes [70]. 

Berberine is an isoquinoline alkaloid found in various medicinal plants, particularly those belonging to the Berberis genus [71]. In animal models of PD, berberine has been shown to have neuroprotective properties. To this end, researchers investigated the sub-cellular localization and blood-brain barrier (BBB) permeability of berberine in a cellular model of PD and a zebrafish PD model using a fluorescently labeled berberine derivative [72,73]. In their study, berberine’s derivative exhibited its anti-PD action by easily passing through the BBB. It also quickly and precisely accumulated in the mitochondria of PC12 cells, according to a subcellular localization analysis. Furthermore, it protected against 6-OHDA-induced cell death, alleviates MPTP-induced PD-like behavior, and increases dopaminergic neuron loss in the brain of PD-affected zebrafish [72].

Embelin is a natural plant product discovered in the fruits of *Lysimachia punctata* (Primulaceae) and *Embelia ribes* Burm (Myrsinaceae) [74]. The findings demonstrate that embelin preserved N27 dopaminergic cells from MPP^+^-induced oxidative stress and apoptosis. Embelin-treated cells had higher levels of pAMPK, SIRT1, and PGC1α, indicating improved mitochondrial biogenesis. Additionally, embelin provided in vivo protection against MPTP-induced decreases in striatal dopamine and the mitochondrial biogenesis pathway [75]. 

Isorhynchophylline is a significant tetracyclic oxindole alkaloid identified from the Chinese herbal remedy *Uncaria rhynchophylla*, used for generations in East Asia to alleviate neurological disorders [76]. Research found that isorhynchophylline substantially decreased MPP^+^-induced apoptotic cell death in PC12 cells and massively diminished MPP^+^-induced endoplasmic reticulum stress responses, both of which are implicated in dopaminergic neuronal death in PD [77]. In another study, a unique alkaloid leonurine, gained from Herba Leonuri (a traditional Chinese medicine) was shown to have a protective effect on mitochondria in PD and blocked oxidative stress [78].

The aforementioned studies implicated the possible application of alkaloids in targeting mitochondria toward preventing and treating PD. Accordingly, alkaloids modulate several signaling/mediators, including ROS, Nrf2-keap1, PGC-1α, pAMPK, α7-nAChRs, and SIRT1.

### 4.3. Terpenes and Mitochondria in PD

Terpenes are a class of plant secondary metabolites possessing neuroprotective effects by regulating mitochondrial-associated pathways. Terpene/terpenoids are made of isoprene units or modified structures. Based on the isoprene unit numbers in their molecule structure, terpene/terpenoids are classified into monoterpenes, sesquiterpenes, diterpenes, triterpenes, and tetraterpenes [79]. 

Carotenoids are abundant in the photosynthetic pigments of plants, and their molecular composition is tetraterpenes (an 8-isoprene polymer). Lycopene, a natural carotenoid, improved mitochondrial structural membrane ability and ATP ratios while inhibited Bax, and the affiliated decline in Bcl-2 levels provided protection against inflammation and oxidative stress in neuronal cells [32]. In another study, lycopene treatment reduced mitochondrial depolarization and cytochrome c release in neurons, suggesting that mitochondrial membrane integrity was preserved [80]. In addition, lycopene reduced MPP^+^-induced mitochondrial ROS generation as well as mitochondrial morphological alterations. In their study, lycopene suppressed MPP^+^-induced opening of the mitochondrial permeability transition (MPT) pore. Lycopene’s preventative benefits towards MPP^+^-induced cytotoxicity could be due to its involvement in enhancing mitochondrial activity. These findings imply that lycopene might be a useful therapy approach for PD [81]. 

Kaur et al. also reported that lycopene inhibited the release of cytochrome c from mitochondria. The findings support the therapeutic potential of lycopene supplements in rotenone-induced PD, which showed beneficial effects in neurodegenerative diseases by modulating oxidative stress [82]. 

Astaxanthin, a keto-structure carotenoid in the microalgae *Haematococcus pluvialis*, prevented mitochondrial malfunction and modulated the production of ROS in an in vivo and in vitro experiment [83]. The docosahexaenoic acid-acylated astaxanthin ester substantially inhibited the progression of PD in an MPTP-induced mice model. It showed a potential role in regulating dopaminergic neuron death in the brain through the mitochondria-mediated route, as well as the JNK and p38MAPK pathways [84].

In SH-SY5Y cells, a pentacyclic triterpenoid called asiatic acid defends against cellular damage and mitochondrial malfunction caused by H2O2 or rotenone. The rotenone-induced increase in voltage-dependent anion channel and mitochondrial membrane potential dispersion was blocked by asiatic acid. Additionally, for the stability of the mitochondria membrane, asiatic acid decreased Bax production and increased Bcl-xL level [85]. In a rotenone-induced cellular model, asiatic acid rescued mitochondria against oxidative damage and apoptosis. Asiatic acid also increased MMP in an isolated mitochondria model, preserving membrane integrity and ATP generation. The findings show that mitochondria are essential in PD, and asiatic acid is a promising option for PD prevention and treatment [86]. 

Andrographolide, a diterpenoid lactone, showed a protective effect on mitochondria by blocking ROS production and modulating the mitochondrial malfunction by balancing the level of ATP in both in vivo and in vitro models. Andrographolide also enhanced the deletion of depolarized mitochondria via a parkin-dependent pathway [87]. In MPTP-exposed mice, andrographolide treatment alleviated behavioral impairments and decreased dopaminergic neuron loss, as well as rotenone-induced cell death in vitro. During PD, andrographolide prevented excessive mitochondrial fission and neuronal damage [88].

Carnosic acid, a diterpene in rosemary, stimulated the mitochondria fusion protein, mitofusin 1 and 2 (MFN1 and MFN2), and optic atrophy 1 (OPA1). It also blocked the fission protein dynamin-related protein 1 (DRP1). Additionally, parkin activated the IKK/NF-κB pathway and increased OPA1 protein production by carnosic acid [89]. In this line, carnosic acid reduced the negative impact of the paraquat model of PD on mitochondrial activity, while reducing ROS/reactive nitrogen species (RNS) generation. Consequently, carnosic acid stimulated Nrf2 in SH-SY5Y cells by modulating the PI3K/Akt pathway, resulting in higher antioxidant enzymes [90]. The MMP was disturbed, the voltage-dependent anion channel 1 (VDAC1) protein was blocked, and cytosolic cytochrome c was increased in SH-SY5Y cells after treatment with 6-OHDA; however, carnosic acid prevented such effects. By stimulating PINK1/parkin-mediated mitophagy, carnosic acid seems to mitigate the neurotoxicity of 6-OHDA [91]. As a result, carnosic acid might be considered a neuroprotective drug for treating PD through mitochondria-associated pathways. 

Boswellic acids, a group of pentacyclic triterpenes, from the genus Boswellia, have been shown to reduce rotenone-induced mitochondrial malfunction by inhibiting the interleukin (IL)-6/signal transducer and activator of transcription-3 (STAT3)/NF-κB signaling pathways [92]. Tormentic acid is a triterpene found in medicinal plants, including *Rosa rugosa* and *Potentilla chinensis* that significantly suppressed ROS’s intracellular aggregation and Bax/Bcl-2 ratio induced by MPP^+^ in SH-SY5Y cells. This effect was primarily exerted through the stimulation of the PI3K/Akt/GSK-3β signaling pathway. Their results show that tormentic acid could be a possible contender for more preclinical research regarding the prevention and treatment of PD [93]. 

Catalpol, an iridoid glucoside extracted from the root of *Rehmannia glutinosa* Libosch, reduced mitochondrial malfunction via decreasing ATP production and enhancing the MPP^+^ level. It also reduced the calcium input of the neuronal cells, ROS buildup, and MPT pores [94,95]. 

**Figure 3:**
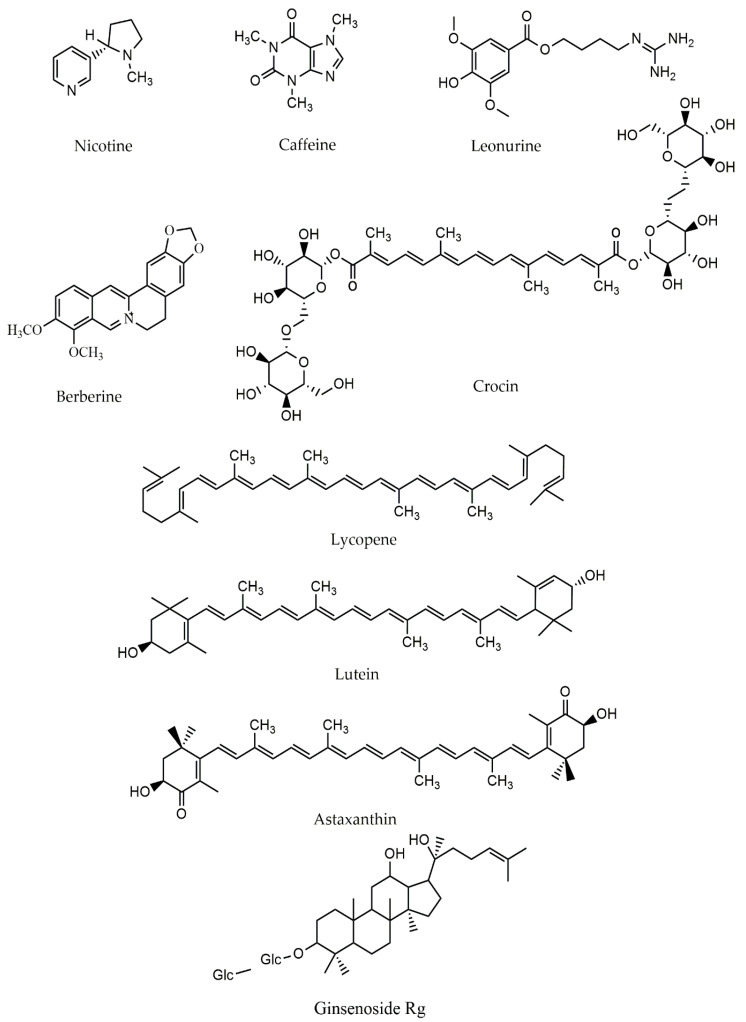
The chemical structure of alkaloids/terpenoids.

Among the other terpenoids, ginsenosides-Rg1, a triterpenoid with a leading role in the efficacy of ginseng, may have a preventive effect on oxidative stress against mitochondrial malfunction in an in vivo experiment [96,97]. Ginseng, the root of *Panax species* (Araliaceae), is a traditional Chinese herbal medicine and a common and highly utilized natural medicine in contemporary times. Ginsenosides are the main pharmacologically active components in ginseng, and they are associated with the majority of ginseng’s actions [98]. Ginsenosides-Rg1 (G-Rg1) have been shown to possess therapeutic benefits with lower in vitro and in vivo toxicity. It also helps with various neurological disorders, particularly progressive neurodegenerative diseases such as PD [99]. Such effects of ginsenosides-Rg1 include enhancing the anti-inflammatory, antioxidation, and antiapoptotic roles. It has also shown the potential to suppress excitotoxicity and Ca^2+^ over-influx into neurons, maintain cellular ATP concentrations, and protect neuronal structural integrity. Excess hydrogen peroxide and other ROS produced by highly reactive iron concentrations can result in mitochondrial malfunction and enhanced dopamine metabolism. Ginsenosides-Rg1(G-Rg1) reduced the number of iron-staining cells in the SN of MPTP-treated mice [96]. It was also discovered that ginsenoside Rb1 (at 10 M) considerably improved cell survival compared to controls by recovering MMP and lessening Ca^2+^ over-influx into the mitochondria, resulting in an increase in the energy produced by mitochondria in primary cultured dopaminergic mouse neurons. In addition, ginsenoside Re was found to repair and offset particular mitochondrial complex IV deficiencies in PINK1-null dopaminergic cell lines by increasing the expression levels of chaperones LRPPRC, Hsp90, and Hsp60 through the restoration of optimum NO signaling. Notoginsenoside R2 (NGR2), a notoginsenoside isolated from *P. notoginseng*, reduced mitochondrial death in SH-SY5Y cells via the MEK1/2–ERK1/2 pathways and suppressing 6-OHDA-induced oxidative stress. Water extract of *P. ginseng* root also inhibited cell death in MPP^+^-treated SH-SY5Y cells by reducing ROS excessive synthesis and inhibiting the mitochondria-dependent apoptotic pathway, as indicated by a reduced Bax/Bcl-2 ratio, cytochrome c release, and caspase-3 activity [100]. The chemical structures of alkaloids/terpenoids are provided in Figure 3. 

Altogether, terpene/terpenoids displayed auspicious anti-PD effects by targeting mitochondria, thereby suppressing complex I–III activity, PINK1/parkin, ROS, Bax, JNK/p38MAPK, IKK/NF-κB, VDAC1, PI3K/Akt/GSK-3β, and IL-6/STAT3/NF-κB. 

Table 1 shows the modulatory effects of plant secondary metabolites on mitochondria and interconnected mediators toward PD prevention/treatment.

## 5. Conclusions

**Figure 4:**
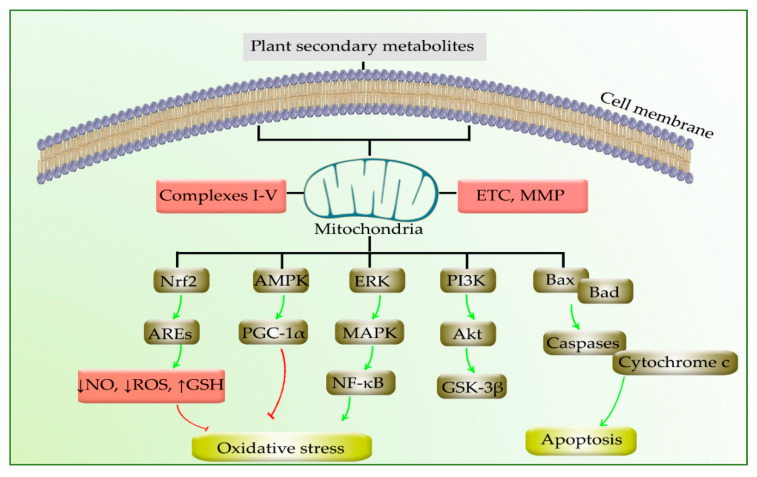
Phytochemicals employ several mechanisms in the treatment of PD passing through mitochondria.

Mitochondria are promising targets in combating several neurodegenerative diseases, especially PD. Since the complexes/enzymes of mitochondria and interconnected oxidative/apoptotic mediators play critical roles in PD, providing multi-target therapies against this disease could open new roads toward finding promising treatments. Plant secondary metabolites are multi-target agents in combating neurodegeneration, employing anti-inflammatory, anti-apoptotic, and antioxidant effects. The potential of phytochemicals in a simultaneous targeting of mitochondria and interconnected mediators could pave the road in treating PD. In this line, the activity of complexes I-V, MMP, PINK1/parkin, ROS, NO, Bcl-2/Bax ratio, PGC-1α, pAMPK, α7-nAChRs, and SIRT1, Nrf2/ARE, JNK/p38MAPK, IKK/NF-κB, PI3K/Akt/GSK-3β, VDAC1, and STAT3/NF-κB are modulated by phytochemicals passing through the mitochondria toward the treatment/prevention of PD (Figure 4). 

Despite their effectiveness, phytochemicals suffer from poor bioavailability, instability/solubility/selectivity, rapid metabolism, and clearance which limit their plasma concentration and therapeutic applications in PD. So, the investigation of appropriate delivery systems (e.g., nanoparticles, micelle, liposome, and solid-lipid nanoparticles) for phytochemicals could counter the limitations in their applications in combating PD by increasing cellular uptake, bioavailability, efficacy and specificity of neuroprotective plant-derived secondary metabolites [79].

The present study highlights the potential of phenolic compounds, alkaloids, terpenoids, and miscellaneous phytochemicals in combating PD passing through mitochondria-associated pathways. Future reports should include additional pre-clinical studies to reveal the critical targeting of mitochondria in PD followed by well-controlled clinical trials to assess phytochemicals as effective treatments. In addition, revealing the direct role of mitochondria and the related beneficial effects of plant secondary metabolites in the pre-clinical and clinical signs of PD would be of great importance. Such reports will further highlight the potential of phytochemicals in the prevention, management, and treatment of PD. 

## Figures and Tables

**Table 1:** Modulating mitochondria by plant secondary metabolites in PD.

Compound(s)	Class	In Vivo/In Vitro Models	Mechanisms and Outcomes	Reference
Pinocembrin	Flavonoid	In vitro: SH-SY5Y cells	Blocking ERK1/2 or silencing of Nrf2	[58]
Naringin		In vivo: adult male Wistar albino rats	↓Rotenone-induced dopaminergic toxicity, ↓mitochondrial function, stability, and bioenergetics in the animals’ SNpc via a Nrf2-mediated path	[54]
Naringenin		In vivo/ In vitro: brains’s neurons of Sprague Dawley rats	↑MMP, ↓ROS via affecting the Nrf2/ARE pathway	[50]
		In vitro: SH-SY5Y cells	↓Mitochondria-related bioenergetics and redox dysfunctions via the Nrf2/GSH pathways	[53]
Hesperidin		In vivo: Mice	Attenuation of mitochondrial complex I, IV, V activity	[62]
Isoliquiritigenin		In vitro: dopaminergic neuronal SN4741 cells	↓Production of ROS as well as the dissipation of MMP and the presence of cytochrome c in the cytoplasm	[53]
		In vitro: dopaminergic neuronal SN4741 cells	↓ROS, ↓NO, ↓JNK and p38 MAPK, ↑Bcl-2/Bax ratio, ↑MMP, ↑BDNF, ↓ cytochrome c, ↓caspase 3 and modulating PI3K/Akt	[101]
Luteolin		In vitro: SH-SY5Y cells	Preventive effect on ROS production and keep the activity of mitochondria in a normal way	[46,60]
Baicalein	Flavone	In vivo/ in vitro: mice/ rat/ SH-SY5Y cells	↓Mitochondrial malfunction in 6-OHDA-induced	[36]
		In vitro: PC12 cells	Modulating ROS production, ↓rotenone-induced apoptotic in PC12 cells via ameliorating the mitochondrial dysfunction	[38]
Apigenin		In vivo: Rat	↑Performance of mitochondrial enzyme	[102]
Chrysoeriol		In vitro: SH-SY5Y cells	↓Toxicity of MPP^+^—induced via PI3K/Akt	[22]
Puerarin	Isoflavone	In vitro: PC12 neuronal cells	↓Toxicity of MPP^+^—induced	[36]
		In vitro: PC12 cells and primary rat midbrain neurons	Regulation of NO-mediated mitochondrial dysfunction	[39]
		In vitro: SH-SY5Y cells	↓Caspase-3 in MPP^+^-induced through modulating the activity of the ubiquitin proteasome system	[40]
Genistein		In vivo: ovariectomized rats	↓Activity of ROS-induced NF-κB/ recovered the production of Bcl-2 mRNA	[18]
Hyperoside	Flavonol glycoside	In vitro: PC12 neuronal cells	↓Mitochondrial apoptotic pathway	[102]
		In vitro: PC12 neuronal cells	↓Mitochondrial apoptotic signaling	[48]
Schisandrin	Lignan	In vivo: Rat cortical cells	↓Ca^2+^, ↓ROS, ↓cytochrome C	[25]
Ellagic acid	Phenolic acid	In vitro: Rat cortical neurons	Modulating ROS production, ↑Bcl-2/Bax	[32]
Ferulic acid		In vivo: Mice	Modulating ROS production by blocking p38MAPK,caspase-3, and COX-2, ↓Bax/Bcl2	[32]
Protocatechuic aldehyde		In vivo/ In vitro: Male C57BL/6 mice/ SH-SY5Y cells	↓ROS, Modulating complex I’s activity in PLK2/p-GSK-3β/Nrf2 pathway	[15]
Caffeic acid		In vitro: SH-SY5Y cells	↓ROS production and keep the mitochondrial activity normal	[54]
		In vivo: male C57BL/6 mice	↓Dopaminergic neurodegeneration and dopamine loss	[55]
Curcumin	Polyphenol	In vivo/ In vitro: mice / PC12 neuronal cell line	↓MPP^+^ toxicity and ROS production	[36,42,59]
Tea polyphenols		In vitro: Human HEK293T and SH-SY5Y cells	Preserving DA neurons via inhibiting DA oxidation, conjugating with DA quinones (DAQ), scavenging ROS, and modulating Nrf2-Keap1 and PGC-1α	[57]
Oleuropein	Secoiridoid	In vitro: PC12 neuronal cell line cells	↓Superoxide anion, ↓complexes I, II, and IV activity, defensive function against MPP^+^	[6]
Hydroxytyrosol	Phenolic compound	In vitro: dopaminergic SH-SY5Y cells	↓Complexes I, II, and IV activity, defensive function against MPP^+^	[6]
Quercetin	Flavonoid	In vivo/ In vitro: aged rats/ mice	↓Apoptosis by downregulation of PI3K/Akt pathway, ↓mitochondrial fission	[42]
		In vitro: SH-SY5Y cells	↓Mitochondrial damage, ↑tyrosine hydroxylase, and mitochondrial controlling proteins	[43]
		In vitro: PC12 cells	↑Mitochondrial quality control, ↓ oxidative stress, ↑mitophagy markers	[44]
		In vivo: adult Sprague-Dawley rats	Repair mitochondrial electron transport abnormalities	[45]
Silibinin	Flavonolig-nan	In vivo: mice	Modulating MMP and mitochondrial activity	[47]
		In vivo:	↓Mitochondrial damage, and strengthening of the oxidative defense system	[48]
		In vivo: Male C57B/6 mice	↓Motor impairment and dopaminergic neuronal degeneration, ↑stability of MMP	[49]
Resveratrol	Stilbene	In vivo/ In vitro: aged rats/ mice	↓Apoptosis by downregulation of PI3K/Akt pathway	[20]
		In vitro: SH-SY5Y cells	↑MMP, ↑mitochondrial dynamics, elongated fragmented mitochondria	[41]
Vanillin	Phenolic aldehyde	In vitro: SH-SY5Y	↓ROS production	[54]
α-Mangostin	Phenolic xanthone	In vitro: SH-SY5Y	Recovered mitochondrial membrane potential and cellular ATP	[102]
Nicotine	Alkaloid	In vivo/ In vitro: rats	↓complex I activity, ↓ROS, Attenuating mitochondrial apoptosis pathway	[32,65]
		In vitro: SH-SY5Y cells	↓MPP^+^ and Ca^2+^-induced mitochondrial high amplitude swelling, ↓cytochrome c release from intact mitochondria	[66]
		In vitro: cultured hippocampal neurons	Mediating mitochondrial dynamics, ↑ IP3 receptor clustering, modulating mitochondria-endoplasmic reticulum communications	[67]
		In vivo/ In vitro: rats	Maintain mitochondrial function	[68]
		In vivo/in vitro: C57BL/6 mouse	↓H2O2-induced astrocyte apoptosis through the mitochondrial route and α7-nAChRs	[69]
Caffeine		In vivo/ In vitro: mice/ dopaminergic neurons	↓ROS production, ↑mitochondrial formation by stimulating Nrf2-keap1 and PGC-1α pathway	[70]
Leonurine		In vivo: mice	Modulating mitochondrial activity	[78]
Berberine		In vitro: SH-SY5Y cells/ mice	↓6-OHDA-induced cell death, ↓MPTP-induced PD-like behavior, ↓dopaminergic neuron loss	[72]
Embelin		In vitro: Rat dopaminergic cell line, N27	↑pAMPK, ↑SIRT1, ↑PGC-1α, ↑mitochondrial biogenesis	[75]
Isorhynchophylline		In vitro: PC12 cells	↓MPP^+^-induced apoptotic cell death, ↓endoplasmic reticulum stress responses	[77]
Lycopene	Carotenoid	In vivo: small mammals	Improving mitochondrial structural membrane ability, ↓Bax	[32,80]
		In vitro: SH-SY5Y cells	↓MPP^+^-induced mitochondrial ROS generation, ↓MPP^+^-induced opening of the mitochondrial permeability transition pore	[81]
		In vivo: Adult male Wistar rats	↓cytochrome c from mitochondria	[82]
Crocin		In vivo: an idiopathic Drosophila	↓complex I_III activity	[103]
Astaxanthin		In vivo/ In vitro: Rat/ SH-SY5Y	↓Production of ROS	[83]
Docosahexaenoic acid-acylated astaxanthin ester			Regulating dopaminergic neuron death in the brain through the mitochondria-mediated route and JNK and p38 MAPK pathways.	[84]
Asiatic acid	Pentacyclic triterpenoid	In vitro: SH-SY5Y cells/ mice	↑Bcl-xL, ↓Bax, ↓H2O2 and rotenone adverse effects	[85]
		In vitro: SH-SY5Y cells	↓ROS, ↓cytochrome c, ↑MMP, preserving membrane integrity and ATP generation	[86]
Catalpol	Iridoid glucoside	In vivo: mouse	↓Mitochondria malfunction via decreasing ATP	[94,95]
Ginsenosides-Rg1	Triterpenoid	In vivo: mice	↓Oxidative stress	[96,97]
Bacosides and Bacopasides	Triterpenoid saponin	in vivo/ in vitro: Drosophila /SH-SY5Y cells	↓Mitochondrial malfunction and oxidative stress, Modulating complex I activity	[104,105]
Andrografolide	Diterpenoid lactone	In vivo/ In vitro: mice brain, N9 mouse microglia (RRID CVCL_0452) cell line	↓ROS, balancing the level of ATP, ↑deletion of depolarized mitochondria via parkin dependent pathway	[87]
			Alleviating behavioral impairments, ↓dopaminergic neuron loss and preventing excessive mitochondrial fission	[88]
Carnosic acid	Diterpene	In vitro: Human SH-SY5Y cells	↑Fusion protein in mitochondria, ↓fission protein activity, ↑OPA1 protein production by parkin in IKK/NF-κB pathway	[89]
		In vitro: SH-SY5Y cells	↓ROS, ↓RNS, ↑Nrf2 via modulating the PI3K/Akt pathway, ↑GSH	[90]
		In vitro: SH-SY5Y cells	↑VDAC1, ↓cytosolic cytochrome c, ↑ PINK1/parkin-mediated mitophagy	[91]
Tormentic acid	Triterpene	In vitro: SH-SY5Y cells	↓ROS, ↓Bax/Bcl-2 ratio through stimulation of the PI3K/Akt/GSK-3β signaling pathway	[93]
Boswellic acids	Pentacyclic triterpene	In vivo: Rats	↓Rotenone-induced mitochondrial malfunction by inhibiting the IL-6/STAT3/NF-κB signaling pathways	[92]
Ginsenosides-Rg	Ginsenoside	In vivo: SN of MPTP-treated mice	Reducing iron-staining cells	[96]
Ginsenoside Rb1		In vivo: mice primary cultured dopaminergic neuron	Recovering MMP and lessening Ca^2+^ over-influx into the mitochondria	[100]
Ginsenoside Re		In vitro: PINK1-null dopaminergic cell line	Repair and offset particular mitochondrial complex IV deficiencies	
Notoginsenoside R2		In vitro: SH-SY5Y cells	↓Mitochondrial death via the MEK1/2–ERK1/2 pathways	
P-ginseng		In vitro: SH-SY5Y cells	↓ROS excessive synthesis and inhibiting the mitochondria-dependent apoptotic pathway	

ARE: antioxidant responsive element, ATP: adenisine triphosphate, Bcl-2: B-cell lymphoma-2, Bcl-xl: B-cell lymphoma-extra-large, Bax: BCL2-Associated X Protein, BDNF: brain-derived neurotrophic factor, DAQ: DA quinones, ERK1/2: extracellular signal-regulated kinas1/2, GSH: glutathione, IKK: IκB kinase, NF-κB: nuclear factor-kappa B, IL-6: interleukin-6, IP3 receptor: inositol 1,4,5-trisphosphate receptor, Keap1: Kelch-like ECH-associated protein 1, MMP: mitochondrial membrane potential, NO: nitric oxide, Nrf2: nuclear factor erythroid 2-related factor 2, pAMPK: phospho-AMP-activated protein kinase, PC12 cells: pheochromocytoma cell, PGC-1α: peroxisome proliferator-activated receptor γ coactivator 1α, PI3K: phosphatidylinositol 3-kinase, Akt: protein kinase B, PLK2: polo-like kinase 2, p-GSK-3β: phosphorylated glycogen synthase kinase-3β, p38MAPK: p38 mitogen-activated protein kinases, RNS: reactive nitrogen species, ROS: reactive oxygen species, SIRT1: sirtuin 1, SNpc: substantia nigra pars compacta, STAT3: signal transducer and activator of transcription 3, 6-OHDA: 6-hydroxydopamine, α7-nAChR: nicotinic acetylcholine receptors.

## Data Availability

Not applicable.
